# Assessment of the Efficacy and Safety of Sublingual Melatonin on Symptom Severity, Quality of Life, and Sleep Disorders in Patients with Irritable Bowel Syndrome

**DOI:** 10.5812/ijpr-156425

**Published:** 2025-02-15

**Authors:** Shabnam Shahrokh, Niloofar Namazi, Mohammad Abbasinazari, Ali Abazarikia, Amir Sadeghi, Arash Mahboubi

**Affiliations:** 1 Gastroenterology and Liver Diseases Research Center, Research Institute for Gastroenterology and Liver Diseases, Shahid Beheshti University of Medical Sciences, Tehran, Iran; 2 Department of Clinical Pharmacy, School of Pharmacy, Shahid Beheshti University of Medical Sciences, Tehran, Iran; 3 Department of Pharmaceutics, Food Safety Research Center, School of Pharmacy, Shahid Beheshti University of Medical Sciences, Tehran, Iran

**Keywords:** Melatonin, Sublingual Tablet, IBS, Trial

## Abstract

**Background:**

Previous studies have demonstrated the efficacy of melatonin in alleviating symptoms of irritable bowel syndrome (IBS) and improving quality of life (QoL).

**Objectives:**

Due to its superior bioavailability, this trial was designed to compare the effects of sublingual melatonin (SL melatonin) with a placebo in alleviating IBS symptoms, enhancing QoL, and addressing sleep disorders.

**Methods:**

The IBS patients were randomly assigned to receive either 3 mg of SL melatonin or a matching placebo for eight weeks. Participants completed the IBS symptom severity score (IBS-SSS), IBS-quality of life 34 items (IBS-QoL 34), and Pittsburgh Sleep Quality Index (PSQI) questionnaires immediately before and after the study period.

**Results:**

A total of 76 patients completed the trial over six months. The results indicated that the severity of IBS symptoms and QoL scores were significantly better in the SL melatonin group compared to the placebo group (P = 0.032 and P = 0.045, respectively). No participants withdrew from the trial due to serious side effects in either the SL melatonin or placebo groups.

**Conclusions:**

Sublingual melatonin may be administered to IBS patients as a complementary treatment to alleviate symptoms and improve QoL.

## 1. Background

Irritable bowel syndrome (IBS) is a common disorder among gastroenterologists, characterized by abdominal pain associated with changes in bowel habits (1). In the United States, IBS has been reported in 7% to 16% of the population, with the highest prevalence among young females (2). Despite the high prevalence of IBS, its management remains challenging. The primary symptoms exhibit significant variability among individuals, and extra-intestinal manifestations, such as sleep disturbances and fibromyalgia, complicate the management of IBS (1). Due to the myriad of issues associated with IBS, patients frequently pursue complementary or dietary therapies to alleviate symptoms. The United States 2012 National Health Interview Survey (NHIS), which covered 13,505 participants, showed that 42% of patients, including those who have suffered from a gastrointestinal disorder, used complementary medicine in the prior year (3). The pathophysiology of IBS remains poorly understood, and it is likely that multiple components are involved (2).

Melatonin is a neurohormone produced by the pineal gland and is also present in the enterochromaffin tissue of the gastrointestinal tract. It has been suggested that melatonin may play a role in the regulation of gastrointestinal motility in IBS, although its mechanism of action remains unclear. Melatonin exerts its function through specific receptors, named melatonin receptors (MT1, MT2, and MT3) (4). Chen et al. published a meta-analysis regarding the efficacy of oral melatonin in the alleviation of IBS symptoms. Their analysis encompasses only four randomized trials, involving a total of 115 patients. They concluded that the administration of melatonin was associated with a greater reduction in overall IBS severity compared to placebo (k = 4, Hedges' g = 0.746, 95% confidence intervals = 0.401 - 1.091, P < 0.001) (5). In all previous trials, the oral tablet of melatonin was administered for the management of IBS, which has a known low bioavailability (below 20%) (6). Attempts to expand the bioavailability of melatonin seem rational to intensify the management of gastrointestinal diseases. Melatonin is a good candidate for sublingual administration because of variability in oral absorption and high first-pass metabolism in the liver (7).

## 2. Objectives

Given the limited number of trials and the lack of focus on evaluating oral melatonin tablets in previous studies, the present study aims to investigate the efficacy, safety, quality of life (QoL), and changes in sleep patterns in IBS patients receiving sublingual melatonin (SL melatonin) compared to placebo.

## 3. Methods

The ethical approval for this double-blind randomized clinical trial was obtained from the ethics committees of the nursing, midwifery, and pharmacy schools of Shahid Beheshti University of Medical Sciences (IR.SBMU.PHARMACY.REC.1402.021). The trial has also been registered in the Iranian Registry of Clinical Trials with the code: IRCT20121021011192N14. Sublingual melatonin (Vana-Darou-Gostar Pharmaceutical Company, Iran) and an identical placebo were produced and packaged in appropriate bottles. After the preparation of the drug and placebo, the clinical trial commenced on March 1, 2024. Eligible participants for the trial included adults over 18 years of age, recruited from the gastroenterology clinics of two centers affiliated with Shahid Beheshti University of Medical Sciences in Tehran, Iran. All participants fulfilled the Rome IV criteria for the diagnosis of IBS (8). To mitigate potential confounding effects of diagnosis on the efficacy of the clinical study medications, eligible participants must have received their IBS diagnosis at least one month prior to entering the trial. Written informed consent was obtained from all volunteers before their enrollment in the trial. Exclusion criteria included pregnant or breastfeeding mothers, individuals with organic gastrointestinal disorders, shift workers, patients using medications with noticeable interactions with melatonin (such as fluvoxamine) or sedative effects (such as tricyclic antidepressants), patients using any dietary or complementary medicines, and patients who required any change in the drug regimen for IBS during the study period.

After selecting eligible patients for the trial, demographic data were collected. Additionally, prior to the administration of SL melatonin or placebo, the volunteers completed three distinct questionnaires: IBS-Severity Symptom Scale (IBS-SSS), IBS quality of life-34 (IBS-QoL34), and Pittsburgh Sleep Quality Index (PSQI). The IBS-SSS Questionnaire represents the initial straightforward approach for monitoring the progression and management of IBS. This instrument evaluates and categorizes pain, distension, bowel dysfunction, and QoL plus global well-being into mild, moderate, or severe categories. Mild, moderate, and severe cases were defined by scores of 75 to 175, 175 to 300, and above 300, respectively (the greatest achievable score is 500). Scores below 75 are considered in remission (9). The IBS-QoL34 Questionnaire is a validated 34-item instrument designed to assess the QoL in patients with IBS. Respondents complete 34 items, which are aggregated to produce a total score ranging from 0 to 100, with lower scores indicating a better QoL for IBS patients (10). Haghayegh et al. have mentioned that the Persian version of the IBS-QoL34 Questionnaire could be a valid and reliable tool to assess patients with IBS (11). The PSQI is a self-rating questionnaire designed to estimate sleep quality over the past month, with scores ranging from 0 to 12, with a higher total score indicating worse sleep quality. It has been validated in the Persian language previously (12).

Upon completion of the baseline questionnaires, eligible patients were randomized using a randomization table to receive either SL melatonin (3 mg daily) or a placebo tablet (one pill daily) for a duration of eight weeks. During the treatment period, patients were evaluated regarding adherence to the medications, and if there was any troublesome adverse effect, the melatonin or placebo was decreased, and the patient was dropped from the trial. At the end of the 8 weeks, participants were interviewed to assess using the three mentioned questionnaires again. Similar to other trials, self-reported data from patients were utilized to assess medication adherence. This assessment involved asking patients whether they had ever forgotten to take any tablets (melatonin or placebo). Patients who missed more than 80% of their doses were considered non-adherent and were subsequently withdrawn from the trial (13).

The statistical package for social sciences (SPSS) version 26.0 (IBM Corp.; Armonk, NY, USA) was used for data analysis. The lowest level of statistical significance was considered at P < 0.05. To determine the sample size for the present trial, the results from Saha et al. (14) were considered, which reported improvements in overall IBS-QoL scores of 43.6% in the melatonin group and 14.6% in the placebo group. Consequently, a minimum of 37 patients per group was required, with an alpha level of 0.05 and a beta value of 80%.

## 4. Results

**Figure 1.**
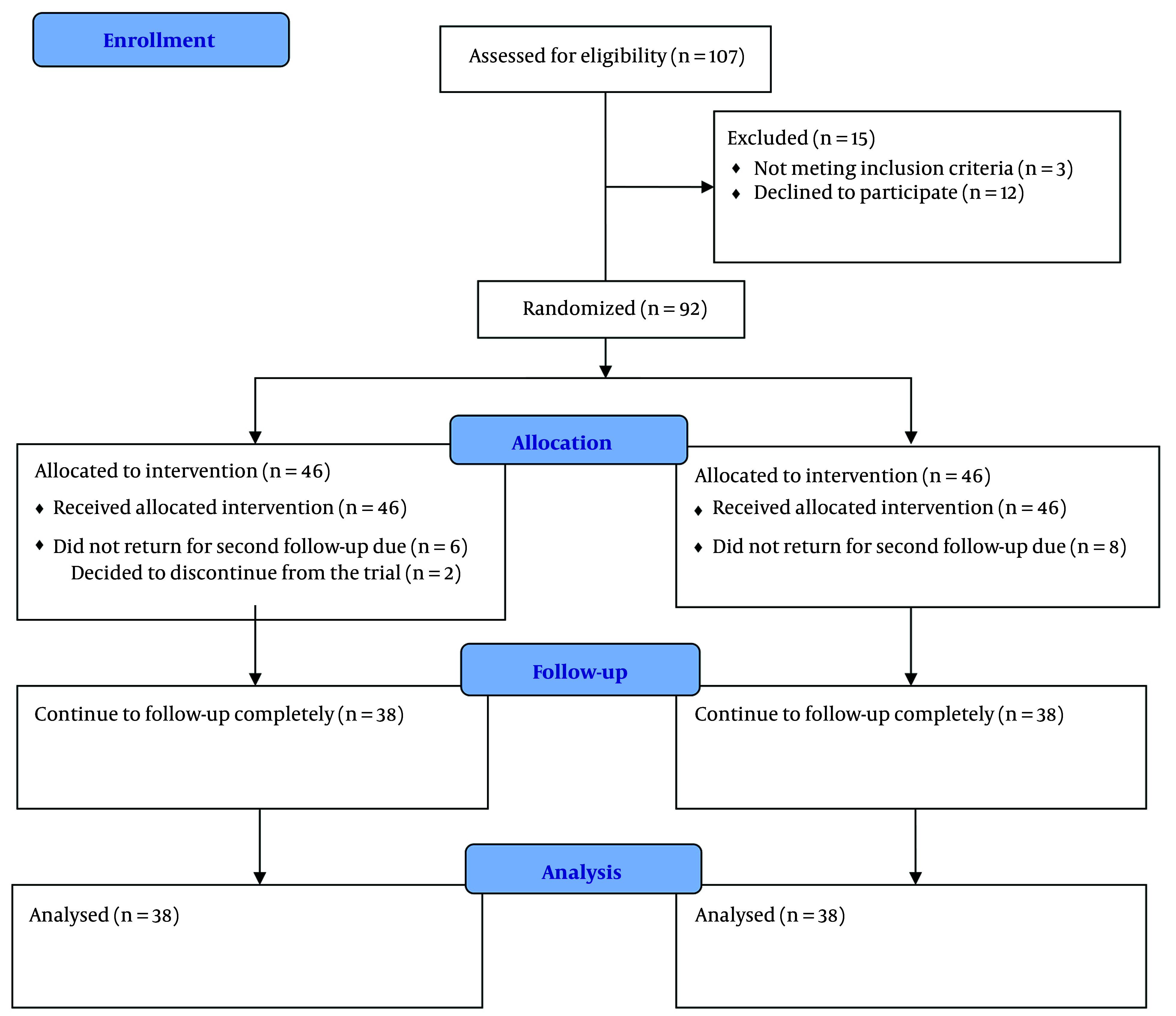
Flowchart of the study

**Figure 2.**
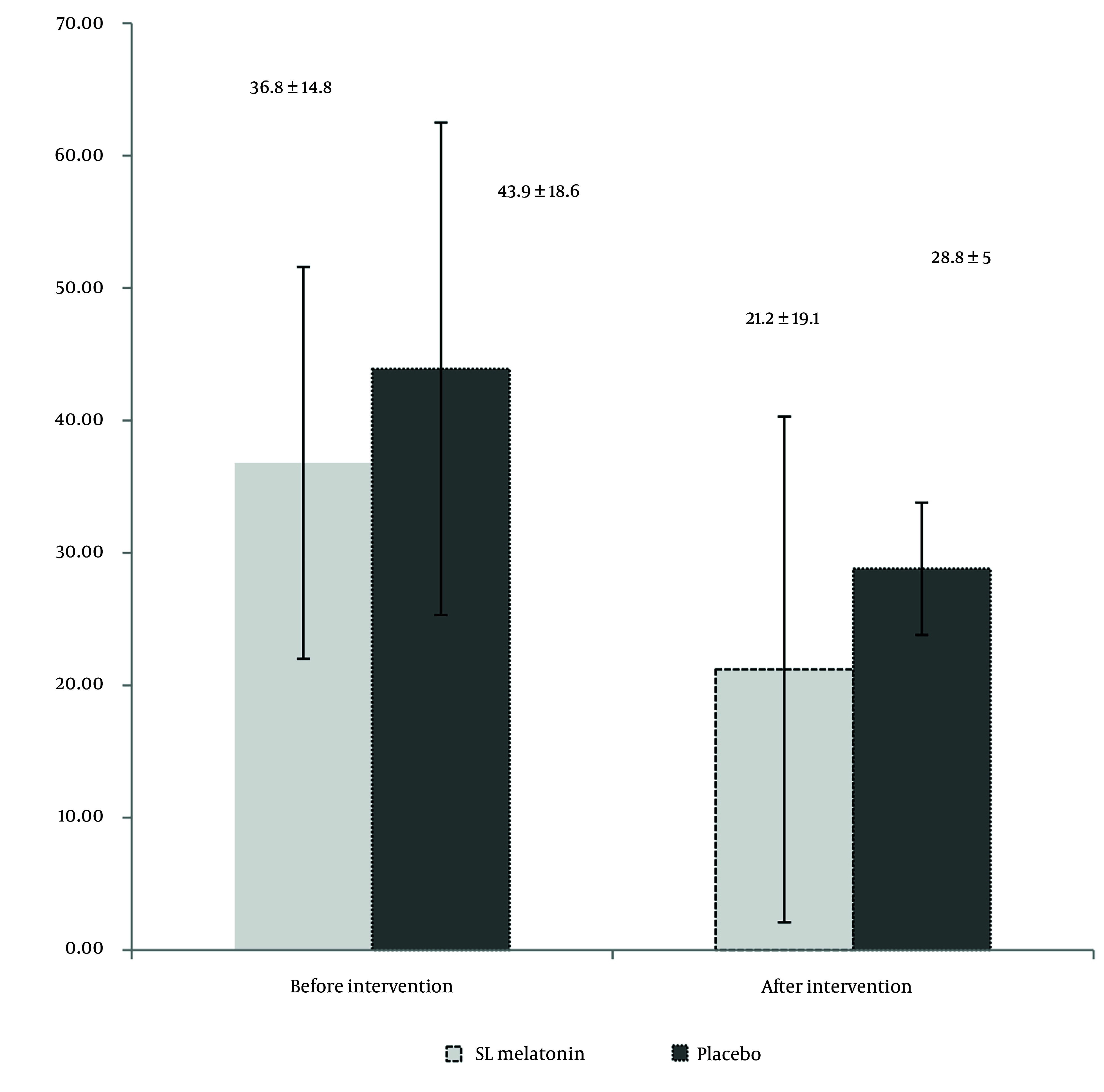
Irritable bowel syndrome quality of life-34 (IBS-QoL34) before and after intervention in two arms

**Figure 3.**
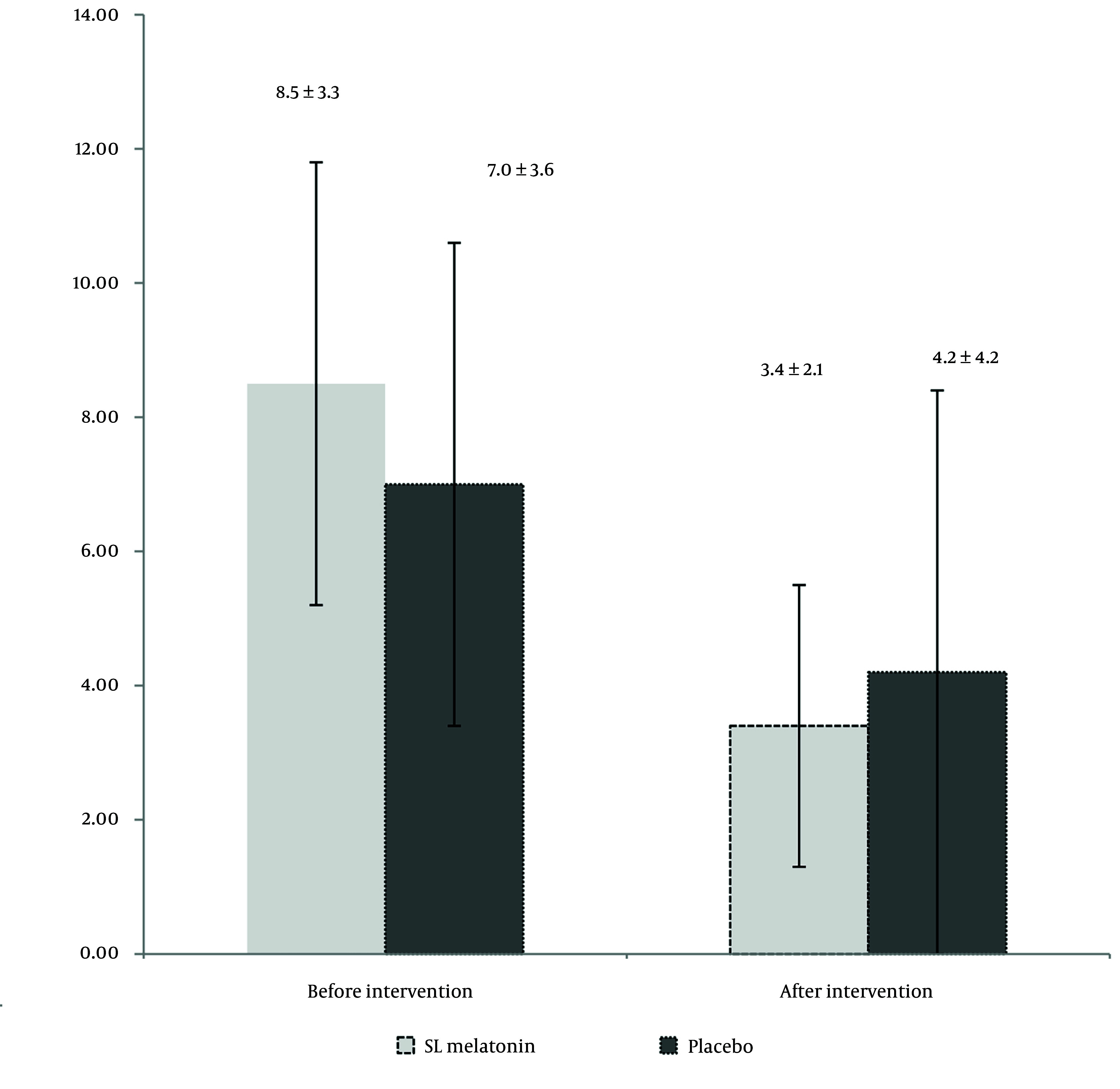
Pittsburgh Sleep Quality Index (PSQI) scores before and after intervention in two arms

During the six months, 76 patients completed the trial. The flow of the trial is shown in Figure 1. Table 1 presents the mean age, sex distribution, IBS type (pain-predominant, constipation-predominant, or diarrhea-predominant), Body Mass Index (BMI), smoking habits, selective serotonin reuptake inhibitors (SSRIs), and anti-IBS medications (anti-pain/anti-constipation and anti-diarrhea) use of the participants. As indicated in Table 1, there are no significant differences between the two groups in terms of demographic and medical characteristics. The IBS-SSS scores in the SL melatonin and placebo arms before and after the intervention are exhibited in Table 2. The pre-intervention IBS-SSS rankings indicated no significant difference between the two groups (P = 0.275). However, post-intervention (after 8 weeks), the IBS-SSS ranking in the melatonin group was significantly lower than that in the placebo group (P = 0.032). In Figure 2, the IBS-QoL34 scores for the SL melatonin and placebo groups are shown before and after the intervention. Although overall IBS-QoL34 did not differ between the two groups before the intervention (P = 0.07), assessment of IBS-QoL34 in the participants revealed a better IBS-QoL34 score in the SL melatonin group than in the placebo group after the intervention (P = 0.045). The PSQI scores of patients are distinctively shown in two groups, before and after the intervention, in Figure 3. Regarding PSQI, there is no difference between the two arms at baseline (P = 0.066) and after eight weeks (P = 0.293).

**Table 1.** Demographic and Medication Data of the Participants ^a^

Variables	Intervention Arm (n = 38)	Control Arm (n = 38)	P-Value
**Age (y) **	38.8 ± 12.5	40.1 ± 12.7	0.688
**Female/male (No.)**	27/11	28/10	0.798
**Type of IBS**			
Pain predominant	13	16	-
Constipation predominant	20	18	0.527
Diarrhea predominant	5	4	-
**BMI (kg/m** ^**2**^ **) **	24. 53 ± 3.18	23.01 ± 3.58	0.430
**SSRIs user **	6 (15.7)	10 (26.3)	0.260
**No smokers **	28 (73.7)	35 (92.1)	0.065
**Anti-abdominal pain medication **	16 (42.1)	25 (65.8)	0.065
**Anti-constipation medication **	8 (21)	12 (31.6)	0.435
**Anti-diarrhea medication **	0 (0)	1 (2.6)	1

Abbreviations: BMI, Body Mass Index; SSRIs, selective serotonin reuptake inhibitors; IBS, irritable bowel syndrome.

^a^ Values are expressed as mean ± SD or No. (%) unless otherwise indicated.

**Table 2.** Irritable Bowel Syndrome Symptom Severity Score of Two Arms Before and After the Intervention

Variables	Before Intervention				After Intervention			
	Intervention Arm	Control Arm	Total	P- Value	Intervention Arm	Control Arm	Total	P-Value
**In remission**	0	0	0	0.275	27	17	44	0.032
**Mild**	3	7	10		7	6	13	
**Moderate**	26	20	46		3	10	13	
**Severe**	9	11	20		1	5	6	
**Total**	38	38	76		38	38	76	

Utilizing the Naranjo score to identify probable adverse drug reactions (ADRs) during the trial (15), no significant difference was observed in the total number of patients experiencing ADRs between the SL melatonin and placebo groups (10.5% vs. 5.2%, P = 0.246). Notably, there were no withdrawals from the trial attributable to ADRs. In the SL melatonin group, reported ADRs included nausea, somnolence, and transient urinary frequency, each observed in three different patients. Additionally, two patients in the placebo group reported experiencing headaches.

## 5. Discussion

To the best of our knowledge, this is the first clinical trial to directly evaluate the efficacy and safety of SL melatonin in ameliorating symptoms of IBS, enhancing QoL, and affecting sleep patterns. The findings of the current study indicate that SL melatonin is significantly associated with improvements in the severity of IBS symptoms as measured by the IBS-SSS (P = 0.032) and overall QoL (P = 0.045). However, no significant improvement was observed in sleep quality (P = 0.293). Our findings are consistent with those of Chen et al. (5), who reported that the administration of oral melatonin tablets is associated with improvements in the severity of IBS and QoL. Although some studies have reported improvements in sleep quality and quantity with melatonin (16), Chen et al. did not observe any significant effects on sleep in IBS patients in their meta-analysis (5). Similarly, our study found no significant differences between SL melatonin and placebo after an 8-week period. Given that this trial represents the initial investigation of SL melatonin in patients with IBS and considering the superior oral absorption of this dosage form compared to conventional melatonin tablets, we opted to utilize 3 mg of SL melatonin in the current study. Administering a higher dose of melatonin might have also resulted in observable improvements in the sleep patterns of the participants.

In the studies conducted by Lu et al. and Saha et al., the specific types of IBS were not identified (14, 16). However, this trial, all types of IBS patients (pain-predominant, diarrhea-predominant, and constipation-predominant) were included. There was no significant difference between the SL melatonin and placebo groups regarding IBS type (P = 0.527). Due to the limited number of patients in each subgroup, it was not possible to conduct a statistical analysis of IBS severity, QoL, and sleep patterns in this study.

Regarding safety, melatonin and SL melatonin appear to be well-tolerated by patients, with no serious ADRs reported in our trial or similar studies. For instance, Saha et al. observed ADRs in three patients (16.66%), with one patient from each group experiencing drowsiness and one patient from the melatonin group reporting decreased libido (14). Similarly, Lu et al. found that only three patients reported symptoms potentially attributable to ADRs of melatonin or placebo. Specifically, one patient in the placebo group experienced a skin rash, another reported vaginal pruritus, and one patient complained of daytime sleepiness with both placebo and melatonin (16). In our trials, the overall rate of ADRs was determined to be 6.6% (7.9% in the SL melatonin group and 5.3% in the placebo group). Based on our results, it appears that the rate of ADRs is lower with SL melatonin compared to melatonin.

Patients with IBS often require multiple medications to manage abdominal pain and bowel habit changes. Consequently, drug interactions are a significant concern for these patients (17). Therefore, careful consideration of potential drug interactions is essential when administering any pharmacologic agents to IBS patients. Melatonin, which has limited drug interactions, can be easily prescribed to IBS patients who are on multiple medications (18). In our trial and similar studies (14, 19), despite the concurrent use of numerous medications by patients, no ADRs due to potential drug interactions were observed.

However, due to the preliminary nature and the limited number of evaluated patients, future randomized clinical trials with larger sample sizes and longer follow-up durations are warranted to support or refute the findings of the current study. In the future, to evaluate the efficacy and safety of SL melatonin in various types of IBS, a large patient sample will be essential. Lu et al. reported a beneficial effect of melatonin on anxiety and depression in IBS patients after 8 weeks of administering 3 mg/day of melatonin compared to placebo (16). Our trial did not assess parameters of anxiety and depression. Therefore, the assessment of psychological parameters such as anxiety and depression is suggested for future studies.

### 5.1. Conclusions

Regarding efficacy and safety, we recommend the administration of sublingual melatonin at a dose of 3 mg twice daily for IBS patients to optimize QoL and alleviate IBS symptoms.

## Data Availability

The dataset presented in the study is available on request from the corresponding author during submission or after its publication. The data are not publicly available due to prevention of a long manuscript.

## Clinical Trial Registration Code:

IRCT20121021011192N14.
